# Multicenter evaluation of BioCode GPP for syndromic molecular detection of gastrointestinal pathogens from stool specimens

**DOI:** 10.1128/jcm.01545-23

**Published:** 2024-02-08

**Authors:** Colleen Knoth, Romney Humphries, J. Kristie Johnson, Anami Patel, Amorce Lima, Suzane Silbert, Jan Vinjé

**Affiliations:** 1 Applied BioCode, Santa Fe Springs, California, USA; 2 University of California Los Angeles Medical Center, Los Angeles, California, USA; 3 Department of Pathology, University of Maryland School of Medicine, Baltimore, Maryland, USA; 4 Le Bonheur Children’s Hospital, Memphis, Tennessee, USA; 5 Tampa General Hospital, Tampa, Florida, USA; 6 Division of Viral Diseases, Centers for Disease Control and Prevention, Atlanta, Georgia, USA; Medical College of Wisconsin, Milwaukee, Wisconsin, USA

**Keywords:** syndromic testing, multiplex PCR, gastrointestinal infection, scalable throughput, diagnostics

## Abstract

Acute gastroenteritis (AGE) is a leading cause of morbidity and mortality worldwide across all age groups that disproportionally affects young children in low- and middle-income countries and immunocompromised patients in high-income countries. Regional outbreaks of AGE are typically detected by traditional microbiological detection methods that target limited organisms and are associated with low sensitivity and lengthy time-to-results. Combined, these may result in repeat testing, imprecise or delayed treatment, and delayed recognition of outbreaks. We conducted a multi-site prospective study comparing the BioCode Gastrointestinal Pathogen Panel (BioCode GPP) for the detection of 17 common bacterial, viral, and protozoan causes of gastroenteritis with reference methods, including stool culture, enzyme immunoassays, pathogen-specific PCR assays, and sequencing. One thousand five hundred fifty-eight residual, de-identified stool samples (unpreserved stool and stool in Cary-Blair transport medium) were enrolled and tested for 11 bacterial, 3 viral, and 3 protozoan pathogens. BioCode GPP and reference methods were positive for 392 (25.2%) and 283 (18.2%) samples, respectively (*P* < 0.0001). In this study, the BioCode GPP and reference methods detected 69 and 65 specimens positive for *Clostridioides difficile*, 51 and 48 for enteroaggregative *Escherichia coli*, 33 and 27 for enterotoxigenic *E. coli*, 50 and 47 for norovirus GI/GII, and 30 and 22 for rotavirus A, respectively. The BioCode GPP showed good positive and negative agreements for each pathogen ranging from 89.5% to 100%, with overall sensitivity and specificity of 96.1% and 99.7%, post adjudication. The BioCode GPP detected >1 pathogens in 49 samples, representing 12.5% of the total 392 positive specimens.

**IMPORTANCE:**

This study highlights performance of a novel technology for timely and accurate detection and differentiation of 17 common bacterial, viral, and protozoan causes of gastroenteritis. Utilizing molecular tests such as the BioCode Gastrointestinal Pathogen Panel may improve the detection of gastrointestinal pathogens and provide actionable results, particularly for patient populations at most risk.

## INTRODUCTION

Acute gastroenteritis (AGE) is a leading cause of morbidity and mortality worldwide, across all age groups (1–4). Globally, it is estimated that 1.53 million people died from infectious diarrheal disease in 2019, with over 500,000 of those fatalities occurring in children under the age of 5 years old. Diarrheal diseases are the third most common cause of death among children in this age group worldwide (4). The burden is greatest in low-income countries with poor sanitation and limited access to clean water (5). In the USA, infectious diarrhea poses a severe and growing risk to vulnerable patient populations, such as those with immune deficiencies, causing prolonged hospital stays and increased mortality (6).

Standard microbiological testing for gastrointestinal pathogens, including culture, enzyme immunoassays (EIA), and microscopy require specialized laboratory skills, and are associated with long time-to-results and low detection rates (7, 8). Single-target PCR assays show improved sensitivity and turnaround time but require multiple PCR assays or sequential testing to detect various infections/coinfections. Since most gastrointestinal (GI) pathogens present with similar symptoms, delayed or lack of identification of the causal agent can significantly impact patient care and lead to community spread (9). Serial or repeat testing is common due to the known poor sensitivity of traditional methods, despite rarely improving diagnostic yield (7, 8). Delayed diagnosis is a major contributor to antimicrobial misuse, increasing the risk of antimicrobial resistance, adverse drug reactions, and the risk of *Clostridioides difficile* infection (10, 11).

As an alternative to conventional methods, multiplex molecular panels configured for syndromic testing have been adopted by clinical labs over the past decade. These multiplex assays can aid in rapid identification of pathogens, enabling rapid outbreak response and infection control in healthcare settings. Despite recent introduction of molecular multiplex pathogen detection platforms, there is a limited choice of platforms for clinical labs with scalable throughput needs (12). Here, we describe the results of multicenter clinical study for BioCode Gastrointestinal Pathogen Panel (BioCode GPP), which simultaneously detects 17 different GI pathogens with scalable throughput.

## MATERIALS AND METHODS

### Specimen collection

For the clinical study, a total of 1,558 leftover, de-identified samples were prospectively collected from patients who underwent stool sample collection for clinical evaluation at four geographically different US sites (California, Tennessee, Maryland, and Florida) between January 2015 and August 2017. Each site contributed an equivalent number of samples for this study (361–400 per site, Table 1). Of the 1,558 specimens, 3 were excluded from all performance analyses because BioCode GPP results were invalid due to a negative internal control signal. Individual reference method failures were excluded from their target-specific performance calculations only.

**TABLE 1:** Specimen enrollment by clinical site and sample type^*a*^

Site	Unpreserved stool		Cary-Blair	Total
	Frozen	Fresh	Fresh	
A	350	50	0	400
B	347	50	0	397
C	263	137	0	400
D	0	0	361	361
Total enrolled	960	237	361	1,558

^*a*^ Stool specimens were collected at four geographically distinct clinical sites in the USA over a 30-month period.

Unformed stool specimens without preservatives were divided into multiple aliquots; culture, EIA, and *C. difficile* reference methods were performed on the freshly collected specimens. The remaining aliquots were either tested fresh on the BioCode GPP and PCR/sequencing reference methods or stored frozen (≤−65°C) if testing could not be performed within 4 days of collection. Alternatively, stool specimens were transferred to Cary-Blair transport medium, aliquoted, and tested according to manufacturer’s instructions [stored at room temperature (15°C–25°C) or refrigerated (2°C–8°C) for up to 4 days].

### BioCode GPP)

BioCode GPP is a qualitative multiplex RT-PCR-based assay that detects and differentiates 17 different gastrointestinal pathogens including bacteria, viruses, and parasites (Table 2). Nucleic acid extraction was performed with either the NucliSENS easyMAG or MagNa Pure 96 extraction systems, and all steps after PCR setup are automated on the MDx-3000. Combined, the test and system allow for scalable batch testing using 96-well microplates.

**TABLE 2:** Comparison of BioCode GPP and reference methods in the positive identification of 17 GI pathogens^*a*^^,^^*b*^

	BioCode GPP results					Reference method results				
Target organism	Unpreserved frozen	Unpreserved fresh	Cary-Blair fresh	Prospective total	Reference method	Unpreserved frozen	Unpreserved fresh	Cary-Blair fresh	Prospective total	Total specimens
*Campylobacter* spp.	19	3	13	35	a	3	1	3	7	1,553
*C. difficile*	0	28	41	69	b	0	27	38	65	597
*Escherichia coli* O157	5	0	2	7	c	2	0	0	2	1,554
STEC	4	2	2	8	d	3	0	0	3	1,520
EAEC	28	1	22	51	e	29	1	18	48	1,542
ETEC	12	6	15	33	e	10	3	14	27	1,543
*Salmonella* spp.	26	5	6	37	c	22	3	5	30	1,554
*Shigella*/EIEC	15	4	4	23	c	5	1	2	8	1,554
*Vibrio parahaemolyticus*	2	1	0	3	a	0	0	0	0	1,555
*Vibrio* spp. (not *V. parahaemolyticus*)	0	0	0	0	a	0	0	0	0	1,554
*Yersinia enterocolitica*	5	1	4	10	a	0	0	0	0	1,554
*Cryptosporidium* spp.	7	1	5	13	e	7	1	3	11	1,542
*Entamoeba histolytica*	0	0	0	0	e	0	0	0	0	1,542
*Giardia lamblia*	8	1	2	11	e	2	0	1	3	1,542
Adenovirus 40/41	10	2	0	12	e	10	0	0	10	1,542
Norovirus GI/GII	43	1	6	50	f	39	1	7	47	1,553
Rotavirus A	27	2	1	30	f	20	1	1	22	1,553
Total positives	211	58	123	392		152	39	92	283	

^*a*^ a, culture; b, FDA-cleared NAAT (Xpert *C. difficile*); c, enrichment culture; d, enrichment culture + FDA antigen test (EIA); e, composite result of PCR/sequencing (Applied BioCode); f, composite result of PCR/sequencing (CDC). STEC, Shiga toxin-producing *E. coli*; EAEC, enteroaggregative *E. coli*; ETEC, enterotoxigenic *E. coli*; EIEC, enteroinvasive *E. coli*. NAAT, Nucleic Acid Amplification Test, CDC, Centers for Disease Control and Prevention.

^*b*^ Unpreserved stool specimens were tested fresh for *C. difficile*, culture, and EIA and were either collected and tested fresh or frozen at ≤−65°C prior to testing with BioCode GPP or PCR/sequencing. Reference method for each pathogen is described in the footnotes. Results for *C. difficile* were tabulated only from fresh specimens.

#### Extraction

One hundred microliters of unformed stool or stool in Cary-Blair transport medium or a loopful of stool (~100 mg) was added to SK38 tubes (Bertin, Rockville, MD) in ~1 mL of S.T.A.R. buffer (Roche, Penzberg, Germany). The prepared specimen was then spiked with 10 µL of BioCode RNA IC (internal process control; provided) and mechanical lysis was performed by vortexing SK38 tubes at high speed for 5 min. Lysate was clarified by centrifugation at 3,500–5,000 rpm for 2 min, and total nucleic extraction was performed with NucliSENS easyMAG (bioMérieux, Durham, NC) with Specific A protocol according to manufacturer’s instructions.

Reflex testing for specimens with initial invalid results was performed by repeating extraction with 50 µL of lysate from the SK38 tube combined with 150 µL S.T.A.R. buffer, instead of initial 200 µL of lysate, with the same easyMAG protocol.

#### PCR setup

Twenty-five microliters PCR reaction was prepared with BioCode Master Mix A (10 µL), BioCode RT Mix (0.5 µL), BioCode GPP Primer Mix (9.5 µL), and 5 µL of extracted sample according to BioCode GPP instructions for use. PCR plate was sealed with pierceable foil and placed in the thermal cycler unit of BioCode MDx-3000 which performs PCR amplification, liquid handling steps, optical detection, and results interpretation via a predefined algorithm.

### Culture and EIA

Culture for bacteria was performed according to each site’s standard of care (SOC) methodologies. Where SOC did not cover some bacteria or include Gram-Negative (GN) broth enrichment, Cary-Blair specimens were sent on ice packs to another site for culture within 4 days of collection. GN broth was used for enrichment of *Salmonella*, *Shigella*/enteroinvasive *Escherichia coli* (EIEC), and Shiga toxin-producing *E.coli* (STEC) or *E. coli* O157 prior to culture on solid medium and EIA for STEC. EIA was used according to each manufacturer’s package insert for detection and differentiation of Shiga toxins 1 and 2.

### Reference molecular assays for norovirus and rotavirus

Viral nucleic acid was extracted from 10% clarified fecal suspensions prepared in phosphate buffered saline using MagMax-96 Viral RNA Isolation Kit (Ambion, Foster City, CA, USA) according to the manufacturer’s instructions on an automated KingFisher extractor (Thermo Fisher Scientific, Pittsburgh, PA, USA) and tested for norovirus GI/GII by real-time reverse transcription-polymerase chain reaction (RT-qPCR) targeting the ORF1/ORF2 junction region (13). Positive samples were typed by conventional RT-PCR targeting 5´-end of the major capsid gene (ORF2) of norovirus following Sanger sequencing of RT-PCR products of expected size (13). Nucleic acids were also tested for rotavirus group A by RT-qPCR and positive samples were confirmed by using a VP6-specific conventional RT-PCR (14, 15).

### Reference PCR/sequencing assays

Twenty-four (16) different PCR/sequencing assays for different pathogens were developed and validated for this study (Table S1). NucliSENS easyMAG (bioMérieux, Durham, NC) was used for extraction of DNA/RNA from stool specimens, and ABI 7500 systems (ThermoFisher, Waltham, MA) was used for real-time PCR amplification with SYBR Green. Briefly, 25 µL PCR reaction was prepared with iTaq SyBr Green Supermix (BioRad, Hercules, CA), assay-specific primers, and 3 µL of extracted sample. After 2 min at 95°C, 40 cycles of PCR was performed with 15 s at 95°C, 40 s at 60°C, and 40 s at 72°C. PCR results were analyzed based on cycle threshold and assay-specific melting temperature ranges. Presumptive positives were verified by bidirectional sequencing with BigDye terminator chemistry and capillary electrophoresis on ABI 3500 Analyzer (ThermoFisher, Waltham, MA). Sequences were analyzed with ABI Sequence Scanner and Lasergene Software (DNASTAR, Madison, WI) to generate the contigs with PHRED quality scores (Q score) ≥20 and <5% ambiguous base calls. NCBI BLAST (17) of each contig was performed to generate Identity to Reference, Query Coverage, and Expected Value. Sequence identification of the target by at least one assay is considered as confirmation of the target pathogen.

### Reference PCR assay for *Clostridioides difficile*

Stool specimens were tested for *C. difficile* with Xpert *C. difficile* (Cepheid, Sunnyvale, CA) according to the manufacturer’s instructions, and only fresh stool specimens were included for clinical performance evaluation.

### Data analysis

Positive and negative agreements for each pathogen compared to reference methods was calculated according to Clinical and Laboratory Standards Institute guidelines (EP09). Clinical sensitivity/positive percent agreement (PPA) was calculated as TP / (TP + FN); TP is true positive or positive by both the reference and BioCode GPP, FN is false negative or negative by BioCode GPP only. Clinical specificity/negative percent agreement (NPA) was calculated as TN / (TN + FP); TN is true negative or negative by both the reference and BioCode GPP, FP is false positive or positive by BioCode GPP only. The exact binomial two- sided 95% CI was calculated by Clopper-Pearson exact method (*Analyze-it* for Microsoft Excel). Chi-squared tests were used to compare positivity rates between the BioCode GPP and reference methods. A chi-squared test was used to assess *C. difficile* PPA differences between fresh and frozen specimens. For both, *P*-values were calculated using MedCalc Statistical Software version 20.218 (18) and *P*-values of <0.05 were considered statistically significant.

### Discordant analysis

Additional testing was performed for specimens with discordant results between BioCode GPP and culture or EIA reference methods by additional PCR/sequencing assays described in Table S2 and/or retesting with a separate aliquot of stool that was stored frozen at ≤−65°C. Results confirmed by PCR/sequencing assays were used as adjudicated results for calculating PPA and NPA. Specimens with discordant results that used PCR/sequencing as the reference method, were characterized by two additional rounds of BioCode GPP and PCR/sequencing with a separate aliquot of stool. Majority of the results (two out three) of PCR/sequencing assays were considered as the truth and used for calculating adjudicated PPA and NPA. No additional testing was performed for discordant analysis of *C. difficile*, norovirus, and rotavirus A.

### Archived positives

Previously characterized, known positive specimens (*n* = 260) were collected from various clinical laboratories in the USA to supplement the limited number of positives obtained from prospective enrollment of specimens. Archived specimen enrollment included *Campylobacter* spp., *E. coli* O157, enterotoxigenic *E. coli* (ETEC), STEC, *Salmonella* spp., *Shigella*/EIEC, *Yersinia enterocolitica, Cryptosporidium* spp., *Giardia lamblia,* and Adenovirus 40/41. Frozen specimens were thawed, aliquoted, and tested with PCR/sequencing assays to confirm the specimen integrity. One specimen was enrolled for both STEC and O157; all others were enrolled for a single target organism. After confirmation of results, known positives were randomized with 152 negative specimens and sent to a clinical site for testing with BioCode GPP. PPA was calculated as indicated above. Archived positives were only tested to confirm presence of the intended target and therefore were not used when calculating negative agreement for other on-panel target organism. NPA was calculated for the 152 negative specimens compared to expected negative status.

### Contrived specimens

Contrived specimens (*n* = 485) for rare pathogens were prepared by spiking multiple strains at a wide range of concentrations, including three times limit of detection, into pre-screened negative specimens (by BioCode GPP). Strains were diluted to 10× the final concentration and spiked into negative stool (final 90% stool). The contrived specimens were positive for *Giardia lamblia, Entamoeba histolytica, Yersinia enterocolitica, Vibrio parahaemolyticus*, or *Vibrio* spp. These specimens were randomized with 127 negative specimens prior to testing with BioCode GPP for a total of 612 specimens. PPA and NPA were calculated compared to expected positive or negative status individually for each target on the panel.

## RESULTS

### Specimen collection and demographics

A total of 1,558 residual, de-identified samples were prospectively collected from hospitalized patients or outpatients for whom a stool sample was submitted to the clinical laboratory for the evaluation of infectious agents, as part of routine care. The study was conducted at four geographically different investigational sites over the 30-month trial period (Table 1). Patients were stratified into four age ranges: ages 5 and younger: 140 patients (9%); ages 6–21: 237 patients (15.2%); ages 22–59: 718 patients (46.1%); and ages 60 and older: 463 patients (29.7%). This population included 778 female patients (49.9%) and 780 male patients (50.1%). With 1,212 inpatients, 77.8% of the population was hospitalized or in the emergency department when samples were collected, while 346 (22.2%) samples were collected from outpatients.

### Prevalence by method

Of the 1,558 specimens, 3 were excluded from all performance analyses because BioCode GPP results were invalid. Individual reference method failures were excluded from their target-specific performance calculations. Prevalence by method is summarized in Table 2. Among the 17 targeted GI pathogens tested, the BioCode GPP detected 392 pathogens compared to 283 by the reference methods (*P* < 0.0001). The most frequently detected pathogens included 69/597 (11.6%) and 65/597 (10.9%) for *C. difficile*, 51/1,542 (3.3%) and 48/1,542 (3.1%) for enteroaggregative *E. coli* (EAEC), 33/1,543 (2.1%) and 27/1,543 (1.7%) for ETEC, 50/1,543 (3.2%) and 47/1,543 (3.0%) for norovirus GI/GII, and 30/1,543 (1.9%) and 22/1,543 (1.4%) for rotavirus group A by the BioCode GPP and reference methods, respectively. While all specimens were processed fresh with standard of care method, 960 unpreserved specimens were frozen prior to testing with the BioCode GPP or PCR/sequencing. In the frozen samples, there were 211 positive results with the BioCode GPP and 152 with reference methods (*P* = 0.002). Within the 237 fresh unpreserved samples, there were 58 and 39 positive results with the BioCode GPP and reference methods, respectively (*P* = 0.054). In the 361 fresh Cary-Blair samples, there were 123 and 92 positive results with the BioCode GPP and reference methods, respectively (*P* = 0.035).

**Fig 1:**
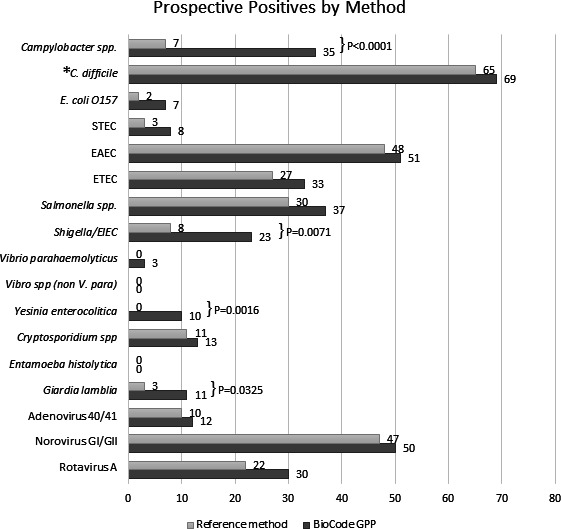
Distribution and number of pathogens detected by BioCode GPP and reference methods for all prospective stool specimens prior to discordant analysis. *P* < 0.05 indicates a difference between the reference method and BioCode GPP. *C. difficile analyzed for fresh only.

Overall, compared to culture methods, more positive results were detected by BioCode GPP (123 and 50, respectively; *P* < 0.0001). In particular, *Campylobacter* spp. was detected in 35 vs 7 specimens by BioCode GPP and reference culture, respectively (*P* < 0.0001; Fig. 1). Twenty of the 29 additional *Campylobacter* positives by GPP (69%) were confirmed by sequencing. Likewise, no *Y. enterocolitica* was detected by culture compared to 10 detected by BioCode GPP (*P* = 0.0016) and 3 of these 10 were confirmed by sequencing. Lastly, more positive results were generated for *Shigella*/EIEC with 23 positives for BioCode GPP and 8 for reference culture (*P* = 0.0071), and 16 of 17 additional Shigella/EIEC positives by GPP were confirmed by sequencing (Table S3).

### Clinical performance

Overall agreements of BioCode GPP with reference methods on prospective specimens were 91.2% PPA and 99.5% NPA prior to and 96.1% PPA and 99.7% NPA after discordant analysis and adjudication. Results for each target organism, pre- and post-adjudication, are shown in Table 3. The results stratified by sample type and storage conditions are shown in Table S3.

**TABLE 3:** Initial and adjudicated results for prospective specimens^*a*^^,^^*b*^

	Initial results (without discordant analysis)							Adjudicated results by PCR/bidirectional sequencing						
	TP	TN	FN	FP	Total	PPA (%)	NPA (%)	TP	TN	FN	FP	Total	PPA (%)	NPA (%)
Target organism	GPP+ Ref+	GPP− Ref−	GPP− Ref+	GPP+ Ref−		TP/ (TP + FN)	TN/ (TN + FP)	GPP+ Ref+	GPP− Ref−	GPP− Ref+	GPP+ Ref−		TP / (TP + FN)	TN / (TN + FP)
*Campylobacter* spp.	6	1,517	1	29	1,553	85.7	98.1	26	1,517	1	9	1,553	96.3	99.4
*C. difficile* (Fresh only)	63	526	2	6	597	96.9	98.9	63	526	2	6	597	96.9	98.9
*E. coli* O157	1	1,546	1	6	1,554	50.0	99.6	6	1,546	0	1	1,554	100.0	99.9
STEC	3	1,512	0	5	1,520	100.0	99.7	8	1,512	0	0	1,520	100.0	100.0
EAEC	43	1,486	5	8	1,542	89.6	99.5	44	1,486	4	7	1,542	91.7	99.5
ETEC	23	1,506	4	10	1,543	85.2	99.3	24	1,506	0	9	1,543	100.0	99.4
*Salmonella* spp.	25	1,512	5	12	1,554	83.3	99.2	34	1,512	4	3	1,554	89.5	99.8
*Shigella*/EIEC	6	1,529	2	17	1,554	75.0	98.9	22	1,529	0	1	1,554	100.0	99.9
*V. parahaemolyticus*	0	1,552	0	3	1,555	N/A	99.8	2	1,552	0	1	1,555	100.0	99.9
*Vibrio* spp. (not *V. parahaemolyticus*)	0	1,554	0	0	1,554	N/A	100.0	0	1,554	0	0	1,554	N/A	100
*Yersinia enterocolitica*	0	1,544	0	10	1,554	N/A	99.4	3	1,544	0	7	1,554	100.0	99.5
*Cryptosporidium* spp.	11	1,529	0	2	1,542	100.0	99.9	13	1,529	0	0	1,542	100.0	100.0
*Entamoeba histolytica*	0	1,542	0	0	1,542	N/A	100.0	0	1,542	0	0	1,542	N/A	100.0
*Giardia lamblia*	3	1,531	0	8	1,542	100.0	99.5	3	1,531	0	8	1,542	100.0	99.5
Adenovirus 40/41	7	1,527	3	5	1,542	70.0	99.7	7	1,527	0	5	1,542	100.0	99.7
Norovirus GI/GII	46	1,502	1	4	1,553	97.9	99.7	46	1,502	1	4	1,553	97.9	99.7
Rotavirus A	21	1,522	1	9	1,553	95.5	99.4	21	1,522	1	9	1,553	95.5	99.4

^*a*^ STEC, Shiga toxin-producing *E. coli*; EAEC, enteroaggregative *E. coli*; ETEC, enterotoxigenic *E. coli*; EIEC, enteroinvasive *E. coli*.

^*b*^ Results for BioCode GPP were compared to reference methods prior to discordant analysis as well as after discordant analysis as adjudicated results.

**Fig 2:**
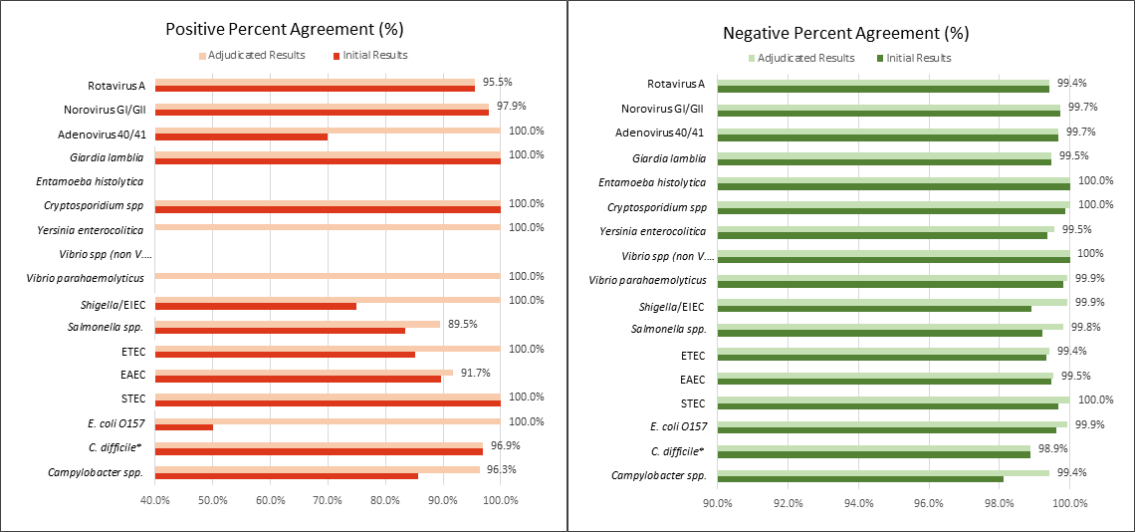
Graphic representation of positive and negative agreements for prospective specimens prior to and after discordant analysis. *C. difficile analyzed for fresh only.

The BioCode GPP demonstrated strong agreement for norovirus GI/GII (97.9% PPA) and rotavirus (95.5% PPA) with the PCR/sequencing assays performed at CDC. PPA for *C. difficile* compared to the Xpert assay (Cepheid) was 96.9% for fresh specimens. Fresh specimens showed a significantly higher PPA than frozen specimens for *C. difficile* (*P* = 0.0388; 94.2%, 65/69 for fresh and 87.7%, 100/114 for frozen), supporting the exclusion of frozen specimens for *C. difficile* from this analysis. Poor PPAs were initially observed for some pathogens with few positives: *E. coli* O157 (1/2: 50%), *Shigella*/EIEC (6/8: 75%), and adenovirus (7/10: 70.0%). However, discordant analysis with PCR/sequencing resulted in significantly improved adjudicated PPAs (Fig. 2): *E. coli* O157 (6/6: 100%), *Shigella*/EIEC (22/22: 100%), and adenovirus (7/7: 100%) (Table 3).

During the prospective study, 2.6% (41/1,558) of samples were invalid due to failure to detect internal control on initial testing. After repeat per instructions for use, the final invalid rate was 0.2% (3/1,558). In addition to the RNA IC for each specimen, an external negative control well is required for each assay run. The BioCode MDx-3000 software suppresses all results if the negative control is invalid for either detection of any target organism or lack of valid RNA IC. During the prospective study performed across the four sites, 2 out of 107 runs produced invalid results for the external negative control well due to unexpected detection of a target organism.

### Polymicrobial co-detections

The BioCode GPP detected a total of 49 samples with polymicrobial co-detections in the prospective clinical study. This represents 12.5% of the total number of positive specimens (49/392). Note that the *C. difficile* positive results from frozen specimens were excluded from the results, which reduced the number of co-detections reported here. Of the co-detections for fresh specimens, 40 were double detections, 8 were triple detections, and 1 was quadruple detection. The most common pathogens in co-infections were EAEC (22/49, 44.9%) and ETEC (18/49, 36.7%). The most common combinations were EAEC with ETEC and *C. difficile* with *Salmonella*.

### Testing archived positive specimens for GI pathogens of low prevalence

Several analytes were either not present or had low prevalence in the prospective study. To supplement the results, 260 archived specimens previously reported as positives were assayed (Table 4). The overall PPA for archived positive samples was 96.0%. Pre-screened negative specimens were randomly mixed with the archived positives in this study; of the 152 negatives, 2 false positive results were observed for an overall NPA of 98.7%.

**TABLE 4:** Positive and negative agreements for archived specimens^*a*^

	Positive agreement		Negative agreement	
Target organism	n/N	PPA (%)	n/N	NPA (%)
*Campylobacter* spp.	38/40	95.0	152/152	100
*E. coli* O157	19/19	100	152/152	100
STEC	30/33	90.9	152/152	100
ETEC	20/20	100	152/152	100
*Salmonella* spp.	29/30	96.7	152/152	100
*Shigella*/EIEC	43/45	95.6	151/152	99.3
*Yersinia enterocolitica*	3/3	100	152/152	100
*Cryptosporidium* spp.	16/19	84.2	152/152	100
*Giardia lamblia*	25/26	96.2	152/152	100
Adenovirus 40/41	26/26	100	151/152	99.3

^*a*^ n/N = detected/total, PPA = Positive Percent Agreement, NPA = Negative Percent Agreement. STEC = Shiga toxin producing *E. coli*, ETEC = enterotoxigenic *E. coli,* EIEC = Enteroinvasive *E. coli*.

### Testing contrived specimens for rare pathogens

Despite enrolling 1,558 prospective specimens and 260 archived positive samples in the study, few positives were detected for pathogens such as *Entamoeba histolytica*, *Y. enterocolitica, Vibrio* species, and *Giardia lamblia*. Therefore, performance was evaluated primarily with contrived specimens (Table 5). PPA was >95% for organisms other than *Vibrio* species which gave 89.5% compared to expected results. Apparent FN specimens were tested by PCR/bidirectional sequencing, and none could be confirmed as positive. It is likely that these samples were prepared below the limit of detection or degraded prior to testing. NPA for all contrived specimens was 99.8%.

**TABLE 5:** Summary of results for contrived specimens^*b*^^,^^*c*^

	Positive agreement^*a*^		Negative agreement	
Target organism	n/N	PPA (%)	n/N	NPA (%)
*Vibrio parahaemolyticus*	88/96	91.7	516/516	100.0
*Vibrio cholerae*	40/47	85.1	518/518	100.0
*Vibrio vulnificus*	42/47	89.4	518/518	100.0
*Yersinia enterocolitica*	95/98	96.9	514/514	100.0
*Entamoeba histolytica*	96/99	97.1	507/513	98.8
*Giardia lamblia*	94/98	95.9	513/514	99.8

^*a*^ Apparent false negative specimens were tested by PCR/bidirectional sequencing, and none were confirmed as positives.

^*b*^ n/N = detected/total, PPA = Positive Percent Agreement, NPA = Negative Percent Agreement.

^*c*^ Pre-screened negative clinical specimens were spiked individually for each pathogen to create contrived specimens.

## DISCUSSION

In this multicenter study, the performance of the BioCode GPP was compared with the reference methods, using 1,558 prospectively collected stool specimens from patients at four sites across the USA over the 30-month trial period and 260 archived positive specimens. The results of this study demonstrated strong agreement overall between the methods, as well as good positive and negative percent agreement by pathogen (Table 3). Negative agreement was >98% for all GI pathogens.

This study demonstrated that the qualitative multiplex RT-PCR-based BioCode GPP yielded significantly more positives compared to reference methods, with an overall sensitivity and specificity of 96.1% and 99.7%, respectively, after adjudication with a low invalid rate (0.2%). This pattern generally holds true for nucleic acid amplification testing techniques (19, 20). Previous studies have also shown that multiplex GI panels provide significantly higher detection rates (22%–74% positive specimens) when compared to standard GI pathogen testing (8%–18% positive specimens) (19, 21, 22). The clinical significance of these additional positive results may vary from institution to institution, depending on patient population, season, and local epidemiology.

Several factors may have contributed to discordant results between the BioCode GPP and reference methods. For example, *Campylobacter* spp., which is the leading cause of foodborne diarrheal infections, was detected in 35 (2.3%) samples by the BioCode GPP and 7 (0.5%) by culture. One possible contributor to this disagreement is that *Campylobacter*, a microaerophilic organism, can be difficult to recover in culture especially if a patient received antibiotic therapy or the sample is not properly stored or transported (16, 23, 24). On the other hand, culture can detect various species of less common *Campylobacter* species (such as *Campylobacter lari* or *Campylobacter upsaliensis*) whereas BioCode GPP detects *Campylobacter coli* and *Campylobacter jejuni*. Similar results observed for *Shigella*/EIEC could also be potentially due to poor recovery by culture.

Storage and testing conditions may have also contributed to some discrepant results. All specimens were tested by culture, EIA, and *C. difficile* reference methods as fresh while ~61% were tested by BioCode GPP after prolonged storage at ≤−65°C. Incidentally, PPA was higher for fresh specimens than frozen specimens, most notably for *C. difficile* (*P* < 0.05). As an anaerobic gram-positive bacterium, *C. difficile* can form spores and is generally not recommended for testing in frozen samples (25). Eliminating frozen specimens still provided sufficient number of positive specimens to determine positive agreement of *C. difficile* in this study, which demonstrated high PPA with a strong confidence interval 96.9 (95% CI 89.5–99.2). As rates of asymptomatic carriage of *C. difficile* can be high in very young children and hospitalized patients, the detection of toxigenic *C. difficile* should be interpreted within the context of guidelines developed by the testing guidelines/policy statements published by the American Academy of Pediatrics, the Society for Healthcare Epidemiology of America, and the Infectious Disease Society of America (26). To aid in diagnostic stewardship effort, the BioCode GPP allows data masking to select targets for reporting based on clinical input and regional infection patterns.

The BioCode GPP detected a total of 49 samples with co-detections in this prospective clinical study. In comparison, co-infections were detected in only eight specimens by reference methods combined (*P* < 0.0001). Diarrhea severity may be influenced by co-infections, making timely detection of co-infections critical to treatment decision-making and patient outcomes (10, 11).

Since stool specimens are known to contain PCR inhibitors, specimens with initial invalid results were reflexed to additional testing. Following reflex testing per instructions for use, the invalid rate was 3/1,558 (0.2%), which is significantly lower than >14% invalid rate for another multiplex molecular assay (27).

Stool culture is a labor-intensive, complex, and low-yield process that can take several days to generate results. With a typical yield rate of under 3% for stool culture, collecting and testing more than one specimen is not uncommon, and patients often are treated with empiric antibiotics, sometimes unnecessarily, pending results (9). An ova and parasite exam is typically ordered when a patient experiences persistent diarrhea or gastroenteritis. These highly specialized and time-consuming tests also have a low sensitivity of under 2% and, therefore, are frequently repeated (28). Other standard laboratory testing methods for GI pathogens, such as direct fluorescent immunoassays and enzyme immunoassays, provide only moderate sensitivity and low yields (8). Likewise, even standard single-target PCR often requires sequential testing to pinpoint the precise causative pathogen (12). There is emerging evidence that patients tested with conventional methods have longer lengths of stay, undergo sequential and/or unnecessary diagnostic testing, receive imprecise therapy, and prolonged treatment courses, when compared to molecular syndromic testing methods such as the BioCode GPP (11, 29).

The BioCode GPP with the MDx-3000 provides definitive results for 17 GI pathogens in about 5 hours with minimal hands-on time. The BioCode GPP and automated MDx-3000 system is FDA-cleared and can process up to 188 patient samples in an 8-hour shift. The system is designed for testing in a 96-well format and can run other panels in parallel allowing mixed batching with other test assays. The platform also provides the ability to mask results for any targets that are not ordered by the clinician, thus enabling customized results. Compared to sample to answer systems that can process individual samples in about an hour, the BioCode MDx-3000 provides with a flexible, scalable throughput option which may benefit high-volume clinical labs during regional outbreaks of AGE (30) or during times of lower volume. The BioCode GPP is validated for use with either raw stool or stool in Cary Blair transport medium. Specimens can be extracted with either the NucliSENS easyMAG (BioMerieux) or MagNA Pure 96 (Roche) automated systems. Testing with the BioCode GPP may reduce time-to-results compared with culture while yielding substantial agreement with the reference methods with significantly improved and high sensitivity and specificity.

### Limitations of the study

Due to the limited number of positive specimens collected for certain organisms during the prospective clinical study, performance characteristics for adenovirus 40/41, *Campylobacter, E. coli O157, Shigella/EIEC, Yersinia enterocolitica*, and *Giardia lamblia* were established with additional retrospective clinical specimens, which provided positive results. Despite enrolling prospective and archived positive samples in the study, few positives were detected for five pathogens. Availability of well-characterized clinical specimens hampered effective evaluation of BioCode GPP for these targets and, consequently, performance was evaluated primarily with contrived specimens (Table 5).

Due to lengthy duration of specimen enrollment, over 60% of the specimens were collected and frozen prior to testing with BioCode GPP. Since culture and some reference methods were performed on fresh specimens, this difference in testing conditions likely contributed to some discordant results, notably for *C. difficile* in frozen specimens. With exclusion of *C. difficile* results from frozen specimens, there were 49 samples with co-detections. Due to lack of clinical information, we were unable to assess if co-infections caused more severe symptoms than comparable patients with single infections.

Finally, the scope of clinical study was limited to evaluation of BioCode GPP in comparison with other diagnostic methods. Additional studies designed to assess the impact of test results on clinical decision making, such as prescribing antibiotics, and effectiveness of patient care may provide useful information on utility and value of multiplex molecular tests such as BioCode GPP.

### Conclusion

While the incidence of AGE mortality has decreased globally over the last quarter century, in part due to the introduction of rotavirus vaccines, it remains one of the largest causes of preventable deaths worldwide. Diagnostic capability to detect and differentiate these pathogens in a timely manner remains a challenge in some clinical settings. This study demonstrated that BioCode GPP, a syndromic gastrointestinal pathogen panel, can significantly increase detection rates, reduce time-to-results and provide marked improvements in sensitivity compared to conventional approaches. Utilizing molecular tests such as the BioCode GPP may improve the detection of gastrointestinal pathogens and provide actionable results, particularly for patient populations at most risk.

## SUPPLEMENTAL MATERIAL

Tables S1, S2, S3 jcm.01545-23-s0001.docx Three supplemental tables for PCR/sequencing reference assays and summary of clinical study results (prospective specimens) stratified by sample type and storage.

ASM does not own the copyrights to Supplemental Material that may be linked to, or accessed through, an article. The authors have granted ASM a non-exclusive, world-wide license to publish the Supplemental Material files. Please contact the corresponding author directly for reuse.
