# Supplementary material for: Multicenter evaluation of BioCode GPP for syndromic molecular detection of gastrointestinal pathogens from stool specimens
Source: J Clin Microbiol. 2024 Feb 8;62(3):e01545-23. doi: 10.1128/jcm.01545-23 (PMC10935640; doi:10.1128/jcm.01545-23)
Supplement: Tables S1, S2, S3 — Three supplemental tables for PCR/sequencing reference assays and summary of clinical study results (prospective specimens) stratified by sample type and storage. [file jcm.01545-23-s0001.docx]

**Table S1.** Summary of PCR/sequencing reference assays

| **Target Organism** | **PCR/sequencing Assay #** | **PCR/sequencing Assay Gene Target** | **PCR/sequencing Assay LoD^a^** | **BioCode GPP LOD^a^** |
| --- | --- | --- | --- | --- |
| EAEC | 1 | aggR | 5.0 x 10^2^ CFU/mL | 1.4 x 10^3^ CFU/mL |
|  | 2 |  | 5.0 x 10^2^ CFU/mL |  |
| ETEC | 1 | LT | 1.0 x 10^4^ CFU/mL | 5.6 x 10^2^ CFU/mL |
|  | 2 | ST1a | 1.0 x 10^4^ CFU/mL |  |
|  | 3 | ST1b | 1.0 x 10^5^ CFU/mL |  |
| Adenovirus 40 | 1,2 | Hexon, Fiber | 1.0 x 10^1^ TCID_50_/mL | 1.0 x 10^-1^ TCID_50_/mL |
| Adenovirus 41 |  |  | 1.0 x 10^1^ TCID_50_/mL | 9.4 x 10^-2^ TCID_50_/mL |
| *Cryptosporidium parvum* | 1 | DNAJ protein | 5.0 x 10^4^ oocysts/mL | 3.1x 10^3^ oocysts/mL |
|  | 2 |  | 5.0 x 10^4^ oocysts/mL |  |
| *Entamoeba histolytica* | 1 | SSU RNA | 5.0 x 10^0^ cysts/mL | 3.1 x 10^-1^ cysts/mL |
|  | 2 |  | 5.0 x 10^0^ cysts/mL |  |
| *Giardia lamblia* | 1 | beta-giardin | 1.0 x 10^3^ cysts/mL | 1.8 x 10^3^ cysts/mL |
|  | 2 |  | 1.5 x 10^3^ cysts/mL |  |

1. LOD values in unpreserved stool

**Table S2.** Summary of PCR/sequencing assays for discordant analysis of targets compared to culture.

| **Target Organism** | **PCR/sequencing Assay Gene Target** | **PCR/sequencing Assay**  **LOD^a^** | **BioCode GPP LOD^a^** |
| --- | --- | --- | --- |
|  |  |  |  |
| *E. coli* O157 | *rfbE* | 3.0 x 10^3^ CFU/mL | 3.3 x 10^3^ CFU/mL |
| STEC (*stx1*) | *stx1* | 3.0 x 10^3^ CFU/mL | 2.5 x 10^3^ CFU/mL |
| STEC (*stx2*) | *stx2* | 1.0 x 10^4^ CFU/mL | 2.5 x 10^3^ CFU/mL |
| EIEC | *ipaH* | 3.0 x 10^3^ CFU/mL | 3.6 x 10^2^ CFU/mL |
| *Shigella* spp. |  | 4.1 x 10^2^ CFU/mL | 4.4 x 10^2^ CFU/mL |
| *Campylobacter coli* | *ceuE* | 9.0 x 10^2^ CFU/mL | 5.6 x 10^1^ CFU/mL |
| *Campylobacter jejuni* | Oxidoreductase subunit | 1.1 x 10^4^ CFU/mL | 7.0 x 10^2^ CFU/mL |
| *Salmonella bongori* | *invA* | 2.2 x 10^4^ CFU/mL | 1.4 x 10^3^ CFU/mL |
| *Salmonella enterica* subsp*. enterica* |  | 4.3 x 10^3^ CFU/mL | 2.2 x 10^3^ CFU/mL |
| *Vibrio cholerae* | *toxR* | 1.2 x 10^2^ CFU/mL | 4.9 x 10^2^ CFU/mL |
| *Vibrio vulnificus* | *vvhA* | 2.0 x 10^3^ CFU/mL | N.D.^b^ |
| *Vibrio parahaemolyticus* | *toxR* | 1.3 x 10^1^ CFU/mL | 1.3 x 10^1^ CFU/mL |
| *Yersinia enterocolitica* | *ail* | 1.5 x 10^3^ CFU/mL | - 1. x 10^3^ CFU/mL |

a. LOD values in unpreserved stool

b. Not tested as part of LOD study for BioCode GPP. Detected at 3x LOD (1.5 x 10^3^ CFU/mL as part of inclusivity study).

**Table S3.** Summary of Clinical Study Results (Prospective specimens) stratified by sample type and storage.

| **Target** | **Specimen Type** | **(n)** | **Positive Agreement** | | **Negative Agreement** | |
| --- | --- | --- | --- | --- | --- | --- |
|  |  |  | **PPA (%)** | **95% CI** | **NPA (%)** | **95% CI** |
| *Campylobacter* spp.^a^ | Cary-Blair (Fresh) | 361 | 2/3 (66.7) | 9.4 - 99.2 | 347/358 (96.9) | 94.6 - 98.5 |
|  | Unpreserved (Frozen) | 955 | 3/3 (100.0) | 29.2 - 100.0 | 936/952 (98.3) | 97.3 - 99.04 |
|  | Unpreserved (Fresh) | 237 | 1/1 (100.0) | 2.5 - 100.0 | 234/236 (99.2) | 97.0 - 99.9 |
|  | All Prospective | 1553 | 6/7 (85.7) | 42.1 – 99.6 | 1517/1546 (98.1) | 97.3 - 98.7 |
| *Clostridium difficile*^b^ | Cary-Blair (Fresh) | 360 | 37/38 (97.4) | 86.2 - 99.9 | 318/322 (98.8) | 96.9 - 99.7 |
|  | Unpreserved (Frozen) | N/A | N/A | N/A | N/A | N/A |
|  | Unpreserved (Fresh) | 237 | 26/27 (96.3) | 81.0 - 99.9 | 208/210 (99.1) | 96.6 - 99.9 |
|  | All Prospective | 597 | 63/65 (96.9) | 89.5 – 99.2 | 526/532 (98.9) | 97.6 – 99.5 |
| *E. coli* O157^c^ | Cary-Blair (Fresh) | 361 | N/A | N/A | 359/361 (99.5) | 98.0 - 99.9 |
|  | Unpreserved (Frozen) | 956 | 1/2 (50.0) | 1.3 - 98.7 | 950/954 (99.6) | 98.9 - 99.9 |
|  | Unpreserved (Fresh) | 237 | N/A | N/A | 237/237 (100.0) | 98.5 - 100.0 |
|  | All Prospective | 1554 | 1/2 (50.0) | 1.3 - 98.7 | 1546/1552 (99.6) | 99.2 - 99.9 |
| Enteroaggregative *E. coli* (EAEC)^d^ | Cary-Blair (Fresh) | 359 | 17/18 (94.4) | 72.7 - 99.9 | 336/341 (98.5) | 96.6 - 99.5 |
|  | Unpreserved (Frozen) | 948 | 25/29 (86.2) | 68.3 - 96.1 | 916/919 (99.7) | 99.1 - 99.9 |
|  | Unpreserved (Fresh) | 235 | 1/1 (100.0) | 2.5 - 100.0 | 234/234 (100.0) | 98.4 - 100.0 |
|  | All Prospective | 1542 | 43/48 (89.6) | 77.3 – 96.5 | 1486/1494 (99.5) | 99.0 - 99.8 |
| Enterotoxigenic  *E. coli* (ETEC)^e^ | Cary-Blair (Fresh) | 359 | 13/14 (92.9) | 66.1 - 99.8 | 343/345 (99.4) | 97.9 - 99.9 |
|  | Unpreserved (Frozen) | 949 | 7/10 (70.0) | 34.8 - 93.3 | 934/939 (99.5) | 98.8 - 99.8 |
|  | Unpreserved (Fresh) | 235 | 3/3 (100.0) | 29.2 - 100.0 | 229/232 (98.7) | 96.3 - 99.7 |
|  | All Prospective | 1543 | 23/27 (85.2) | 66.3 – 95.8 | 1506/1516 (99.3) | 98.8 - 99.7 |
| Shiga toxin-producing *E. coli* (STEC)^f^ | Cary-Blair (Fresh) | 361 | N/A | N/A | 359/361 (99.5) | 98.0 - 99.9 |
|  | Unpreserved (Frozen) | 922 | 3/3 (100.0) | 29.2 - 100.0 | 918/919 (99.9) | 99.4 - 100.0 |
|  | Unpreserved (Fresh) | 237 | N/A | N/A | 235/237 (99.2) | 97.0 - 99.9 |
|  | All Prospective | 1520 | 3/3 (100.0) | 29.2 – 100.0 | 1512/1517 (99.7) | 99.2 - 99.9 |
| *Salmonella* spp.^g^ | Cary-Blair (Fresh) | 361 | 4/5 (80.0) | 28.4 - 99.5 | 354/356 (99.4) | 98.0 - 99.9 |
|  | Unpreserved (Frozen) | 956 | 18/22 (81.8) | 59.7 - 94.8 | 926/934 (99.1) | 98.3 - 99.6 |
|  | Unpreserved (Fresh) | 237 | 3/3 (100.0) | 29.2 - 100.0 | 232/234 (99.2) | 96.9 - 99.9 |
|  | All Prospective | 1554 | 25/30 (83.3) | 65.3 – 94.4 | 1512/1524 (99.2) | 98.6 - 99.6 |
| *Shigella*/ EIEC^h^ | Cary-Blair (Fresh) | 361 | 1/2 (50.0) | 1.3 - 98.7 | 356/359 (99.2) | 97.6 - 99.8 |
|  | Unpreserved (Frozen) | 956 | 4/5 (80.0) | 28.4 - 99.5 | 940/951 (98.8) | 97.9 - 99.4 |
|  | Unpreserved (Fresh) | 237 | 1/1 (100.0) | 2.5 - 100.0 | 233/236 (98.7) | 96.3 -99.7 |
|  | All Prospective | 1554 | 6/8 (75.0) | 34.9 – 96.8 | 1529/1546 98.9 | 98.3 - 99.4 |
| *Vibrio parahaemolyticus^i^* | Cary-Blair (Fresh) | 361 | N/A | N/A | 361/361 (100.0) | 99.0 - 100.0 |
|  | Unpreserved (Frozen) | 957 | N/A | N/A | 955/957 (99.8) | 99.3 - 99.97 |
|  | Unpreserved (Fresh) | 237 | N/A | N/A | 236/237 (99.6) | 97.7 - 99.99 |
|  | All Prospective | 1555 | N/A | N/A | 1552/1555 (99.8) | 99.4 - 100.0 |
| *Vibrio* spp. (not *parahaemolyticus*) | Cary-Blair (Fresh) | 361 | N/A | N/A | 361/361 (100.0) | 99.0 - 100.0 |
|  | Unpreserved (Frozen) | 956 | N/A | N/A | 956/956 (100.0) | 99.6 - 100.0 |
|  | Unpreserved (Fresh) | 237 | N/A | N/A | 237/237 (100.0) | 98.5 - 100.0 |
|  | All Prospective | 1554 | N/A | N/A | 1554/1554 (100) | 99.8 - 100.0 |
| *Yersinia enterocolitica*^j^ | Cary-Blair (Fresh) | 361 | N/A | N/A | 357/361 (98.9) | 97.2 - 99.7 |
|  | Unpreserved (Frozen) | 956 | N/A | N/A | 951/956 (99.5) | 98.8 - 99.8 |
|  | Unpreserved (Fresh) | 237 | N/A | N/A | 236/237 (99.6) | 97.7 - 99.99 |
|  | All Prospective | 1554 | N/A | N/A | 1544/1554 (99.4) | 98.8 - 99.7 |
| *Cryptosporidium* spp.^k^ | Cary-Blair (Fresh) | 359 | 3/3 (100.0) | 29.2 - 100.0 | 354/356 (99.4) | 98.0 - 99.9 |
|  | Unpreserved (Frozen) | 948 | 7/7 (100.0) | 59.0 - 100.0 | 941/941 (100.0) | 99.6 - 100.0 |
|  | Unpreserved (Fresh) | 235 | 1/1 (100.0) | 2.5 - 100.0 | 234/234 (100.0) | 98.4 - 100.0 |
|  | All Prospective | 1542 | 11/11 (100.0) | 71.5 – 100.0 | 1529/1531 (99.9) | 99.5 - 100.0 |
| *Entamoeba histolytica* | Cary-Blair (Fresh) | 361 | N/A | N/A | 359/359 (100.0) | 99.0 – 100.0 |
|  | Unpreserved (Frozen) | 948 | N/A | N/A | 948/948 (100.0) | 99.6 - 100.0 |
|  | Unpreserved (Fresh) | 235 | N/A | N/A | 235/235 (100.0) | 98.4 - 100.0 |
|  | All Prospective | 1542 | N/A | N/A | 1542/1542 (100) | 99.8 - 100.0 |
| *Giardia lamblia*^l^ | Cary-Blair (Fresh) | 359 | 1/1 (100.0) | 2.5 – 100.0 | 357/358 (99.7) | 98.5 - 100.0 |
|  | Unpreserved (Frozen) | 948 | 2/2 (100.0) | 15.8 - 100.0 | 940/946 (99.4) | 98.6 - 99.8 |
|  | Unpreserved (Fresh) | 235 | N/A | N/A | 234/235 (99.6) | 97.7 - 99.99 |
|  | All Prospective | 1542 | 3/3 (100.0) | 29.2 - 100.0 | 1531/1539 (99.5) | 99.0 - 99.8 |
| Adenovirus 40/41^m^ | Cary-Blair (Fresh) | 359 | N/A | N/A | 359/359 (100.0) | 99.0 - 100.0 |
|  | Unpreserved (Frozen) | 948 | 7/10 (70.0) | 34.8 - 93.3 | 935/938 (99.7) | 99.1 - 99.9 |
|  | Unpreserved (Fresh) | 235 | N/A | N/A | 233/235 (99.2) | 97.0 - 99.9 |
|  | All Prospective | 1542 | 7/10 (70.0) | 34.8 – 93.3 | 1527/1532 (99.7) | 99.2 - 100.0 |
| Norovirus  (GI/GII)^n^ | Cary-Blair (Fresh) | 354 | 6/7 (85.7) | 42.1 - 99.6 | 354/354 (100.0) | 99.0 - 100.0 |
|  | Unpreserved (Frozen) | 956 | 39/39 (100.0) | 91.0 - 100.0 | 913/917 (99.6) | 98.9 - 99.9 |
|  | Unpreserved (Fresh) | 236 | 1/1 (100.0) | 2.5 - 100.0 | 235/235 (100.0) | 98.4 - 100.0 |
|  | All Prospective | 1553 | 46/47 (97.9) | 88.7 – 100.0 | 1502/1506 (99.7) | 99.3 - 99.9 |
| Rotavirus A | Cary-Blair (Fresh) | 361 | 1/1 (100.0) | 2.5 - 100.0 | 360/360 (100.0) | 99.0 - 100.0 |
|  | Unpreserved (Frozen) | 956 | 19/20 (95.0) | 75.1 - 99.9 | 928/936 (99.2) | 98.3 - 99.6 |
|  | Unpreserved (Fresh) | 236 | 1/1 (100.0) | 2.5 - 100.0 | 234/235 (99.6) | 97.7 - 99.99 |
|  | All Prospective | 1553 | 21/22 (95.5) | 77.2 – 99.9 | 1522 /1531 (99.4) | 98.9 - 99.7 |

a – *Campylobacter* spp. The 1 false negative compared to reference culture method was tested by bidirectional sequencing and confirmed as positive. The 29 false positives compared to the reference culture method were tested by bidirectional sequencing, and 20 of 29 confirmed as positives.

b – *Clostridium difficile*: The 2 false negatives compared to a FDA cleared NAAT produced high Ct values (Ct ≥ 35), and the 6 false positives had low MFIs which indicate that these samples are low positives. P value = 0.0388 when comparing PPA for fresh vs frozen specimens ( P < 0.05). *C. difficile* must be tested fresh.

c - *E. coli* O157. The one false negative compared to the reference culture method was tested by bidirectional sequencing and could not be confirmed as positive. The 6 false positive samples compared to the reference culture method were tested by bidirectional sequencing, and 5 of 6 confirmed as positives.

d – EAEC. The 5 false negatives compared to bidirectional sequencing were tested by 2 additional rounds of sequencing; 4 of the 5 confirmed as positive. 2 of the 8 false positives could not be repeated due to low sample volume. For the remaining samples, 5 of 6 were not detected by addition rounds of sequencing.

e – ETEC. The 4 false negatives compared to bidirectional sequencing were tested by 2 additional rounds of sequencing; none were confirmed as positives. 1 of the 10 false positives could not be repeated due to low sample volume. Of the remaining 9 false positives, 8 were not confirmed as positives by an additional round of sequencing.

f – STEC. The 5 false positive samples compared to the reference culture method were tested by bidirectional sequencing, and all 5 were confirmed as positives.

g – *Salmonella* spp. The 5 false negative samples compared to the reference culture method were tested by bidirectional sequencing and 4 of 5 confirmed as positives. The 12 false positives compared to reference culture method were tested by bidirectional sequencing and 9 of 12 confirmed as positives.  *Salmonella* species observed in the clinical study were: 8 *S.* enterica groups B-D, 9 S. enterica (untyped), and 17 Salmonella species (untyped).

h – *Shigella*/EIEC. The 2 false negatives compared to the reference culture method were tested by bidirectional sequencing and were not confirmed as positives. The 17 false positive samples compared to reference culture were tested by bidirectional sequencing and 16 of 17 confirmed as positives.

i - *Vibrio parahaemolyticus*. The 3 false positive samples compared to the reference culture method were tested by bidirectional sequencing, and 2 of 3 confirmed as positives.

j – *Yersinia enterocolitica.* The 10 false positive samples compared to the reference culture method were tested by bidirectional sequencing, and 3 of 10 confirmed positives.

k – *Cryptosporidium* spp. The 2 false positive samples compared to bidirectional sequencing were confirmed as positive by 2 additional rounds of sequencing.

l – *Giardia lamblia*. The 8 false positive samples compared to bidirectional sequencing were not confirmed as positive by 2 additional rounds of sequencing.

m - Adenovirus 40/41. The 3 false negatives compared to bidirectional sequencing were tested by 2 additional rounds of sequencing and a FDA-cleared NAAT; none were confirmed as positives by either method. On initial testing these were detected by only one sequencing assay and all had high Ct values, which indicate that they are low positives. The 5 false positives were not confirmed as positives by an additional round of sequencing.

n – Norovirus GI/GII. The 1 false negative compared to bidirectional sequencing produced a high Ct (37).
